# Treatment of Dental Caries, Complicated with Disorders of the Pulp and Peridental Membrane

**Published:** 1852-04

**Authors:** Robert Arthur


AMERICAN JOURNAL

OF

DENTAL SCIENCE.

Vol. II. NEW SERIES—APRIL, 1852. NO. 3

### ARTICLE I.

*Treatment of Dental Caries, Complicated with Disorders of the Pulp and Peridental Membrane.* By ROBERT ARTHUR, D. D. S.

### NO. IV.

IN the last number of this series of papers, I considered those cases in which the caries had not yet progressed so far as to deprive the pulp of any part of its bony covering, but had approached so nearly to this part of the tooth as either to exercise an injurious influence upon it, or to render it liable to become exposed in the course of the performance of the ordinary operations for the arrest of this disease. Considering it of great importance to preserve the pulp alive, and in healthy condition, I advised and indicated such a course of treatment as, in my estimation, would enable this object to be accomplished.

I come now to the consideration of those cases in which the pulp is actually laid bare, and the affected tooth, in such condition, that a filling inserted in the ordinary way, without previous preparation or unusual precaution, or even in the manner directed in the last number, alluded to above, would give rise to painful symptoms and injurious consequences.

To avoid circumlocution, I shall say, that in this condition the pulp is exposed: by which term, I mean what, indeed, is generally understood both in and out of the profession, that the pulp is, to some extent, deprived of its bony covering.

The propriety of treating teeth after the pulp may have become exposed, with any view to their permanent preservation, has long been questioned. Doubts have always been entertained of the feasibility of saving the pulp alive after it is once exposed: and it has been repeatedly asserted, that any attempt to save a tooth after the "nerve" had been "destroyed," would be sure eventually to result in injury to the patient. It was alleged that, when the pulp loses its vitality, the entire tooth is "dead," and that nature, from that time forth, would make unceasing efforts to get rid of it, and that it would ultimately be expelled from the jaw. It will not be necessary to waste time in showing, at any great length, the fallaciousness of this opinion, for a large majority of the most intelligent dental practitioners of the present day, agree that teeth may be preserved for many years after having been deprived of their pulps. It may be well, however, for the benefit of those who are just coming into the profession, to take some notice of these objections: this I shall do in few words.

The teeth, it is well known, derive their supply of blood-vessels, nerves, &c. from two sources, viz. from the investing membrane of the root, and from the pulp occupying the internal cavity. By far the more important of the two is the former; indeed, a highly intelligent member of the profession (Dr. Trenor, of New York,) contended, some years ago, that the teeth are entirely dependent upon the peridental membrane for nourishment, and that the pulp is of no particular value after it has fulfilled its office of forming the tooth. In support of this view, he stated that he had seen teeth from which the pulps had been removed and filled, by Dr. Hudson, which had remained as good and useful as any of the rest of the teeth for ten, fifteen, and twenty years. Whatever may be the amount of truth in the position taken by the gentleman here mentioned, every practicing dentist is aware of this fact, (which alone is sufficient effectually to refute the assertion that teeth, from which the pulp has been removed, are entirely deprived of their vitality,) viz. that fangs, upon which artificial teeth are set, by what is known as the pivoting method, will remain in perfectly healthy condition for many years. I have known instances in which they have remained for thirty years, without giving rise to morbid action of any kind. It is well known, too, that the roots of the molar teeth, the crowns of which have been destroyed by caries, remain for years without producing the slightest irritation until they are, by a well known process, expelled from the jaws, and it will be always found, when they are extracted, no matter how slight may be their adhesion, that the peridental membrane, at some point, retains perfectly its vitality.

No other evidence is necessary to any one, however slightly he may be acquainted with the laws of physiology and pathology, to convince him that teeth are not entirely deprived of vitality when the pulp is taken away, than the fact just stated, that such teeth or roots do remain in the mouth without causing irritation. For it is well known that foreign substances, with few exceptions, cannot remain embedded in any of the living tissues without causing inflammation in such degree as to insure their speedy expulsion.

I do not propose to give more than this slight passing notice to such objections to the propriety of such treatment of teeth in this condition, because, as I have just stated, few, if any, intelligent practitioners of dental surgery, at this day, deny the practicability and usefulness, in some cases, of the process here alluded to, for the preservation of teeth after the pulp may have become exposed.

At the present time, the only question at issue relates to the best means of treating such cases. Two methods are proposed for this purpose, the objects of which are: 1st, Preserve the pulp uninjured. 2nd, To remove it entirely both from the pulp cavity and roots, and fill the place occupied by it with some substance not capable of being decomposed by the fluids of the mouth.

To effect the preservation of the pulp after it becomes exposed, the only means now relied upon by its advocates is, I believe, so to fill the cavity of decay as to avoid bringing the filling into contact with the pulp. This is accomplished either by placing over the pulp, at the place where it is exposed, a cap of gold plate, or so managing the filling as to leave a vacant space between it and the pulp.

The practice has been warmly advocated, and as strongly condemned. In my own hands, as I have already stated—although I am free to confess that I have practiced it in but few cases—it has not proved so satisfactory as to justify me in recommending it from my own experience. Professor Harris, of the "Baltimore College of Dental Surgery," who has had large experience in this operation, takes strong ground in its favor; and in a late number of the Journal, gives the results of a series of observations, running through a period of five years, which places this operation in a more favorable light than I have ever before seen it presented.

But I do not propose to go into a discussion of the relative merits of these two methods of treating the cases in question. It is not, indeed, necessary to my purpose: for, even if it be granted that the process just alluded to is the better one, a question which I am now disposed to leave each practitioner to decide for himself, its more sanguine advocates do not deny that there are cases of frequent occurrence, in which it is not advisable to attempt it, and that, in these cases, there is no alternative left between the sacrifice of the affected tooth and the extirpation of the pulp.

In some cases at least, then, in the present state of our knowledge, this operation becomes indispensable, and the best methods of performing it should be freely canvassed.

It may be well here to show why it is that when the destruction of the vitality of the pulp cannot be avoided, its entire removal is considered necessary, and what is expected to be accomplished by this and by filling the place occupied by it. This I shall endeavor to do.

The entire removal of the pulp, when this operation is attempted at all, is necessary for two important reasons. 1st.—To prevent inflammation of the peridental membrane, alveolar abscess, and its consequences. 2d.—To prevent the subsequent discoloration of the teeth so treated.

When the integrity of the body of the pulp is once even partially broken up, either as a consequence of diseased action, or in an attempt to destroy its vitality, it is my opinion that it rarely if ever again properly performs its functions. Its circulation no longer goes on normally, the blood which continues to be brought through the canal of the root, and through the healthy to the diseased part is no longer returned to the system, but escapes in the form of serum, lymph, healthy or unhealthy pus. As long as any portion of the pulp, retaining vitality, remains either in the pulp cavity or the canal of the roots, to invite blood into the interior of the tooth, this must occur. When it is removed and excised at the extreme end of the root, it may easily be conceived that these parts recover their healthy action, and in a little time, the blood formerly brought into the dental cavity is diverted by the anastomosing vessels into some other channel.

It is obvious, that as long as the matter formed in the pulp cavity or roots, finds means of ready exit, no pain or trouble of any kind will occur; but as soon as its egress is obstructed, it will accumulate till the vacant space is filled, and as the blood continues to be forced into the cavity, will give rise to a train of painful symptoms and consequences, with which every dentist is so well acquainted as to render a description of them unnecessary.

It does not follow, however, that in such cases, even if the obstruction is a permanent one, that teeth in this condition, will inevitably be lost. For, if the subject has the fortitude and patience to bear for a time the painful consequences which ensue, relief from pain will finally come in the form of an alveolar abscess, and the forming matter will once more find a way of escape. In time, the remaining portion of the pulp will be entirely deprived of its vitality, the cause of irritation, if so much of the integrity of the periosteum about the apex of the root is not destroyed, so as to leave the bone bare, and consequently a cause of unceasing irritation, will pass away, the abscess may heal up, and the tooth become apparently healthy. But in such cases there is generally unhealthy action about the parts for many years, and a permanent discharge is kept up.

A great deal more could be said here, but as it properly belongs to another branch of my subject, the cause of and treatment of disorders of the peridental membrane, I will now pass it by.

2d. The entire removal of the pulp is necessary to prevent the discoloration of teeth treated in this way, because the decomposed tissues, and the discharged matter, will gradually pass into the bony substance of the crown, by which this part of the tooth will become, as is well known, much discolored, and eventually, in some cases, quite black. This is seen in a marked degree, in cases where, by a blow, the vessels passing to the pulp are destroyed. As a consequence of the death of the pulp, this discoloration of the teeth is so well known that we are many times asked by patients to whom this operation is proposed, in the case of teeth near the front of the mouth, whether they will not turn black. To accomplish this object, it will readily be seen, however, that the entire removal of the pulp from the minute portions of the canals of the root, is not so necessary as for the object just indicated, for if the pulp cavity and one-third of each root were filled, if no other untoward consequences were to be anticipated, the infiltration of the small remains of the pulp into the dental bone, would be effectually prevented.

The utility of the other part of the process: that of filling with gold, the place formerly occupied by the pulp, is not so clear. It is contended that after the pulp is entirely removed, fluid of some kind escapes from the point at which the vessels have been excised, and that it is important that such fluids should be excluded from the canal in the root and the pulp cavity, because they are liable, eventually, to bring about the destruction of the tooth treated, by being decomposed and passing, by infiltration, through the parieties of the root, and thus reaching the peridental membrane. But even if this do not occur, such fluids, if it be granted that they do escape at this point, and find their way into the root, must effect the discoloration of the tooth by passing into the bony substance of the crown.

It is very easy, indeed, to conceive that a small quantity of fluid may be thrown off from these excised vessels, which may pass into the pulp cavity. If space is left for such fluids to accumulate and be retained until they become putrid and acrid, they must be productive of injurious consequenses to the surrounding parts, either by passing through the root, as above indicated, or filling completely the pulp cavity, and fang, they are thrown back upon the surface from which they have come, producing there all the consequences of the contact of such matters with living tissue. It is supposed that such fluids will again be absorbed, if they are immediately thrown back upon the surface from which they have come, which, it is maintained, will be accomplished by filling the root to the very apex, so as to leave an exceedingly small vacant place only, for the accumulation of such matter.

Such are some of the reasons given for the necessity of the perfect filling of the root in these cases, by those who have most extensively practiced this operation.

But it is by no means satisfactorily determined that any discharge does take place from the excised vessels and nerves, without the root, which are supposed to recover their healthy condition. And I am bound to say that observation has not satisfied me that it is the case. It is certainly safer and better, however, to fill carefully the larger roots of such teeth as are treated in the way indicated. But I cannot discover the utility of this practice in the case of those minute, hair-like canals in the external root of the upper molar teeth, which cannot, indeed be filled unless they are first enlarged ; and this is a process which requires so much time and labor, as so much to increase the cost of the operation as to place it out of the reach of most persons.

The utility, and in some cases the necessity, of the operation just indicated is now admitted by the better portion of the profession. But there is still amongst these a difference of opinion as to the best manner in which it should be performed. It is still a question whether it is better to remove the pulp with instruments alone, or first, by some means, to deprive it of vitality. For this purpose it is scarcely necessary to state that arsenic in some form, has for some years been extensively and almost exclusively used. It is well known, too, that this agent will certainly, uniformly, and completely, destroy the vitality of every portion of living tissue with which it is brought into contact, and if in the case of the dental pulp, it is employed with proper precaution, will effect this object without producing pain of consequence.

It has lately been proposed to apply the actual cautery by means of galvanic electricity. The instrument proposed for the purpose, has already been described in the "Journal." For the destruction of the vitality of the body of the pulp, this instrument could no doubt be readily and effectually applied, but when it is remembered, that a *loop* must be formed of the platinum wire used, it will at once be seen how impossible it would be to pass this into the canal of the root, in those cases in which it will scarcely admit an instrument a great deal larger than a single hair. This instrument, as beautiful and effectual as it seems on first presentation, is, I think I can show to be, of little value for our purpose. It is certainly so if it cannot be applied to the pulp in the contracted canals of the roots, for a better method of destroying the vitality of the body of the pulp, is by the application of arsenious acid; it is better, because it is certain in its effects, painless, and, I think I can show, harmless.

The dental pulp is endowed with a high degree of sensibility; when, from any cause it becomes inflamed, this natural sensibility is exalted to such a degree as to render the slightest touch of any foreign substance, a cause of the most excruciating pain. This has rendered it exceedingly desirable that some means should be used first to destroy its vitality, and thus remove this extreme sensibility before attempting to cut it out. Arsenic, as we have stated above, has been found most effectual for this purpose, and at the present time, is used to the exclusion of every other substance known generally to the profession.

It has been strongly contended, however, that arsenic produces results more or less injurious in every case in which it is used, and it is regarded by some as always advisable to remove the pulp with instruments, regardless of the pain inflicted, rather than to use arsenic for the purpose of destroying the vitality of the pulp. This is the question which now divides the opinions of those who practice this operation.

It is not denied that the immediate effects of arsenic, applied for this purpose, are extremely satisfactory, but it is stated that the final good result of the operation is endangered by its use. The manner in which this occurs, has never been clearly stated, to my knowledge—certainly not in any publication which has come within my reach. But, from what I can gather from various sources, it seems to be the impression that particles of arsenic are absorbed at once, and carried to the peridental membrane, producing irreparable injury to those important parts; or that after the lapse of some time, they, in some way, reach this membrane, and thus cause their injurious results.

Now, as I have stated, I have seen no account of the manner in which arsenic used for this purpose is supposed to be likely to produce injurious results. It has been stated by practitioners of high standing, it is true, that in their practice, it had not proved satisfactory, but had been more or less injurious. But what is their experience, when unsustained by argument, to me, when it conflicts with my own experience? and what is the value of their opinion in this regard, stated to be based simply upon their own experience, without further rational evidence, to the profession at large, when it comes into conflict with the experience of many others of equally high standing with themselves. It is not at this time sufficient to say, that in any one's opinion, a certain course is not a good course to be pursued. Let us therefore examine this question rationally on its own merits.

It has been vaguely stated, from time to time, as we have already intimated, that arsenic applied for the purpose of destroying the vitality of the pulp, would sooner or later, bring about the destruction of the tooth so treated—1st, by being absorbed and carried through the root, and so attack the peridental membrane. 2d. By passing laterally through the parieties of the roots; and 3d, of remaining after the pulp is removed, and in course of time finding its way to the investing membrane.

These are all the reasons I have ever heard of to account for the alleged injurious results of arsenic applied for this purpose.

Let us examine these statements consecutively, for it is important that we should come to a right understanding of this matter.

First—Can arsenic be absorbed, and by this means reach the investing membrane through the agency of the pulp?

Arsenic, we are told, by high authority, (See Liebig's Agricultural Chemistry, chap. xiv, on Poisons, Contagions, Miasms,) is not absorbed when brought into contact with living tissues. It combines with the surface of the organ to which it may be applied, destroys its vitality, and of course the power of absorption, which is a vital function. Where the quantity of arsenic applied is so small that every atom enters into combination with a corresponding number of atoms of the living tissue, if the organs have vigor enough, it is thrown off with the tissue which has been destroyed by its action. If there is an excess of arsenic, it may reach the vital portion of the tissue below the surface, by imbibition or capillary attraction.

If this authority is of any value—and even if it is not, the known action of arsenious acid upon the living tissues, is sufficient to bring any reasoning mind to a similar conclusion—it must be plain that arsenic cannot reach the investing membrane of the root by absorption.

It may, however, if it be applied in excess, and allowed to remain sufficiently long, gradually penetrate so far by passing through the destroyed portion in the way indicated, to the vital portion, and so on till it has passed through the root.

But, although it must be granted, that injurious effects may occur in this way, it can easily be shown that arsenic, applied in the usual manner for destroying the vitality of the pulp, is not allowed to remain long enough to produce the effects indicated. It is generally allowed to remain twenty-four hours only. That this is not sufficiently long to enable it to make its way through the fang, by way of the canal, is abundantly proved by this fact: a small portion only of the pulp, on the removal of the arsenic applied, will be found deprived of vitality; it is probable that the vitality of the body of the pulp will be destroyed, whilst the portion in the fang will be found to retain all its vitality, and to display an increased degree of sensibility. Now, this must be regarded as incontestible evidence, that the arsenic applied has not passed through the root, because its invariable effect is to destroy entirely the vitality of any portion of living tissue with which it comes into contact. It may be said, that the fact that the part alluded to, retains its vitality, is no evidence that some of the arsenic has not reached it, but only that it has not had time to effect its destruction; but this objection is shown to have no weight, because these parts retain their sensibility for many days, and even weeks, as all who have practiced this operation must have found.

If the views and facts here presented, be correct, it is clear to me that no injury can reasonably be supposed to follow the use of arsenic in the ordinary way for this purpose, by absorption, and that when allowed to remain the usual time, no bad effects can follow. I should be glad to be shown it, if I am wrong in the position I have taken. I am sincerely in the search of truth, and am not anxious to establish any of my own opinions, unless they are founded upon this basis.

Second—Do the particles of the arsenic applied, pass laterally through the parieties of the fang?

Third—Do particles of arsenic remain after the pulp is removed, and ultimately reach the investing membrane, and thus, after a lapse of time, give rise to injurious consequences?

These queries may be answered together.

The only way in which this is possible, after the pulp is carefully and entirely removed, will be by passing through the bony substance itself by capillary attraction. That this is possible when it is remembered that the dental bone is porous, and the arsenious acid is in a minute state of division, as it is when rubbed up with creasote, which, I believe, is capable of dissolving it, cannot be denied. But that it does not occur, is sufficiently evident from a little careful observation, and a little careful consideration of the phenomena, which would present themselves if this did occur. The arsenious acid is generally applied in the same manner, dissolved in creasote, generally in the same quantity, and allowed to remain the same length of time. Making due allowance for the difference in density of the teeth of different individuals, we must look for some degree of uniformity in the results of arsenic used in this way. It would take a certain length of time for the arsenic to pass through the root, and we cannot suppose the difference of density usually found to exist in the teeth of different persons, would make any very great difference in this respect. The time required to allow the arsenic to pass by this means through the bone, reasoning from the analogous case of the same substance applied to the bone of a living tooth near the pulp, could not be very long. A month, we should think, would be ample time. Yet, after years have elapsed, we observe no consequence of the kind. And it must be remembered that if arsenic found its way through the root in this way, that its peculiar effect upon the investing membrane would with certainty display itself, and would with certainty result in the destruction of such parts of the investing membrane which it touched. I find it impossible, then, in this view of the case, not to conclude that none of the arsenic after it is applied in the usual way, is left behind after the pulp has been removed and the pulp cavity and canal in the root washed out, or if any should remain, it is so inconsiderable as to do no harm.

I think I have shown that we have no reasonable ground to suppose from the nature of arsenic and the manner in which it affects living tissues, that it can exert any injurious influence when applied for the purpose here indicated.

I am anxious that this point should be established, for, unless we can use arsenic or some agent effectual for the purpose accomplished by it, our usefulness in this way will be extremely limited. The removal of the pulp, unless it is first deprived of vitality, is, in all cases, a painful operation, in most cases excruciatingly so. It is a painful operation to remove the pulp from the incisor and canine teeth, but it can be done with great rapidity, and although the pang of pain produced is, in most cases, exceedingly severe, it is so soon past, that many persons can be induced to bear it. But when the pulp is to be removed from the contracted fangs of the molar teeth, in which it is often exceedingly difficult to find even the openings of the fangs, the operation is so excruciatingly painful, that I am sure not one patient in fifty, who have passed through my hands, would be willing to submit to it. I have already endeavored to show why it is important that the pulp should be effectually removed.

I am aware that gentlemen have declared that the pain attendant upon the operation is inconsiderable, but this is inconceivable, I must confess, to me, unless it is performed by some method with which I am unacquainted.

I have thus far been endeavoring to show that arsenious acid used in the manner in which it is usually and generally employed in the profession at the present day, cannot reasonably be expected to do the injury which has been attributed to its agency. It must be remembered that many, if not most of those gentlemen who have opposed its use, have been extremely cautious how they have applied it, using a very small quantity (the twentieth part of a grain) and allowing it to remain a very short time, (from twelve to twenty-four hours.) This last point is strongly insisted upon, as also the importance of avoiding a second application to the same tooth.

Now, I am prepared to go still further, and to declare my belief from careful observation of the effects produced by it in my hands, that it may not only be applied more than once, but be allowed to remain much longer than this, not only without injury, but with great advantage. It is true, that I do not use it in the form of arsenious acid, and this may make an important difference. Of this I will say more in the proper place.

Before proceeding farther, I beg leave to offer in proof of these assertions, and the above reasoning, a record of some of the cases which have passed through my hands in the course of the past six years. I have not kept a record of all my cases. Those which I now present have not been selected. In almost every case here cited, the arsenious acid has been applied more than once, in some cases as often as five or six times.

Since January, 1846, the time at which I began to record these cases, I find marked down the treatment of seventy-seven teeth in this way.

Of these, 19 were incisor, or canine teeth.

31 were bicuspides.

20 were first and second molar teeth.

7 were dentes sapientiæ.

Of the incisor and canine teeth, 18 were of the upper, and 1 of the lower jaw.

Of the 31 bicuspid teeth, 22 were of the upper, and 9 of the lower jaw.

Of the 20 first and second molar teeth, 14 were of the upper, and 6 of the lower jaw.

Of the dentes sapientiæ, all were lower teeth.

Of these cases, I have, from time to time, covering several years, seen twenty-six. I have not been obliged to extract one of these. In one only, which has come under my observation, has alveolar abscess occurred, and this by no means an aggravated case. It formed in about two years after the operation was performed. The tooth treated was an inferior dens sapientiæ, and an unfavorable case, because, from a variety of causes, it was impossible to perform the operation thoroughly. Another tooth filled for the same lady at the same time, a second inferior molar, was in a perfectly healthy condition, presenting no trace of inflammation at the time I had an opportunity of examining the other one.

Of these cases, I have seen six which had been treated in this manner, some considerably over two years, and all over eighteen months, these were in a perfectly healthy condition, no trace of peridental inflammation being apparent.

In some of these twenty-six cases, seen several months after the operation, there had been occasional slight attacks of inflammation of the peridental membrane, evinced by more or less tenderness to pressure, but which always passed away without giving rise to serious consequenees.

I have not felt obliged, as I have stated, to extract a single tooth I have treated in this way, during the five years I have been in practice in Washington. I have treated more cases than I have recorded, and the patients are where I should be sure to hear from them, if trouble occurred. That not many of them have passed into the hands of other practitioners, I may safely conclude, because most of them still send their friends to me, when they desire the services of a dentist.

It will be observed from this statement, that I have seen, from time to time, one-third of the cases of this kind which have come under my treatment. Out of these, but one case can be said to have failed, and this was evidently traceable to an imperfect operation. But even this cannot be regarded as a failure, for many such cases of abscess occur, which are completely cured by the removal of the irritating cause, probably in this instance some portions of pulp remaining in the smaller parts of the canals of the roots, and they frequently heal spontaneously.

What I principally desire to establish, by the recorded cases offered here, is that the bad effects attributed to the action of arsenic applied for the purpose of destroying vitality of the pulp, do not in reality follow its use. For if any portion of the arsenic applied were to reach the external membrane, which it could only do by passing laterally through the parieties of the root, for all ingress through the root must be prevented by the filling, it would certainly and surely, and speedily produce effects which would make the extraction of the tooth necessary. The same would be the result if any portion, however small, were allowed to remain in the canal above the filling, its effects would, indeed, be more quickly displayed.

I know that such records of general results as I have presented here, are not very highly valued in the profession: for men, the most honest in their intentions, are so apt to see things in a favorable light when they wish to do so. After theories are formed, we are so apt almost unconsciously to make facts bend so as to establish their truth, and facts stated as having been seen in this light, are justly regarded with many grains of allowance. But the cases I have presented, if they are true, and I affirm that no bias or prejudice could lead me to make, knowingly, a single misstatement or exaggeration, even if they do not convince the sceptical of the permanent utility of the operation (for it may, with truth, be said that time enough has not yet elapsed to test its full value,) it will at least make good the point for which I am now contending, and will show that the operation advised is productive of results sufficiently desirable to warrant its practice. In the course of the treatment of the cases of which I have here presented, a mere meagre outline of general results, I have learned many instructive lessons. I have in many of these cases recorded a great deal that was interesting and useful to me at the time, and which has led me to conclusions which I hope I may be able to state in such a manner that they will be useful to others. I know well, now, that if I could have had the same directions to guide me in the course of my own practice, in this way, I should have been saved much trouble and my patients much pain.

